# Evaluation of molecular and serological testing for imported urogenital schistosomiasis screening in a referral tropical medicine centre in Barcelona, Spain

**DOI:** 10.1186/s13071-025-06832-w

**Published:** 2025-05-30

**Authors:** Patricia Martínez-Vallejo, Alejandro Mediavilla, Aroa Silgado, Francesc Zarzuela, Lidia Goterris, Carles Rubio Maturana, Nuria Serre-Delcor, Inés Oliveira-Souto, Fernando Salvador, Joan Joseph-Munne, María Luisa Aznar, Diana Pou, Begoña Treviño, Israel Molina, Javier Sotillo, Elena Sulleiro

**Affiliations:** 1 Microbiology Department, Vall d’Hebron University Hospital, PROSICS Barcelona, Barcelona, Spain; 2 Universitat Autònoma de Barcelona (UAB), Barcelona, Spain; 3 Centro de Investigación Biomédica en Red de Enfermedades Infecciosas (CIBERINFEC), Instituto de Salud Carlos III, Madrid, Spain; 4 Infectious Diseases Department, International Health Unit Vall D’Hebron-Drassanes, Vall d’Hebron University Hospital, PROSICS Barcelona, Barcelona, Spain; 5 Centro Nacional de Microbiología, Instituto Salud Carlos III, Majadahonda, Madrid, Spain

**Keywords:** Urogenital schistosomiasis, *Schistosomahaematobium*, Urine, Molecular diagnostics, Real-time PCR, Serological diagnosis

## Abstract

**Background:**

Schistosomiasis, a major neglected tropical disease, is caused by *Schistosoma* spp. It is estimated that more than 200 million people are affected worldwide, mostly in Africa. The gold standard diagnosis of urogenital schistosomiasis (UGS) is the microscopic visualisation of *Schistosoma haematobium* eggs in concentrated urine; however, its sensitivity is low. This study aimed to evaluate the effectiveness of molecular and serological testing for imported UGS screening in asymptomatic sub-Saharan migrants in a non-endemic setting.

**Methods:**

A retrospective cross-sectional study between November 2021 and December 2022 was conducted by collecting demographic, clinical and laboratory data from the medical records of migrants from endemic areas screened for UGS at the International Health Unit Vall d’Hebron-Drassanes, Barcelona, Spain. Urine samples were analysed by real-time PCR for *S. haematobium* DNA and by microscopy for egg detection. Serum samples were tested using a serological assay based on enzyme-linked immunosorbent assay (ELISA). UGS was confirmed by a positive result in real-time PCR and/or microscopy, while possible UGS was defined as a case with only a positive serological result.

**Results:**

A total of 604 patients were included in this study; 32 out of 604 (5.3%) urine samples were positive for *S. haematobium* by real-time PCR and/or microscopy examination (confirmed UGS cases). *Schistosoma haematobium* DNA was detected in 28/604 (4.6%) urine samples, while eggs were visualised in 24/604 (3.9%), with 12 discordant cases between both techniques.

Real-time PCR demonstrated a sensitivity of 83.3%, a specificity of 98.6%, and a kappa value of 0.76. Serology was performed in 529/604 cases and exhibited lower specificity, 70.87% (kappa value 0.26). Other laboratory parameters such as leukocyturia, microhaematuria, eosinophilia and elevated IgE were significantly associated with UGS diagnosis.

**Conclusions:**

Real-time PCR proved to be more sensitive than microscopy for diagnosing imported UGS in non-endemic settings, with minimal discordance between methods. The serological test exhibited very low specificity and high sensitivity rates, suggesting its usefulness as a screening test among high-risk populations in non-endemic settings.

**Graphical Abstract:**

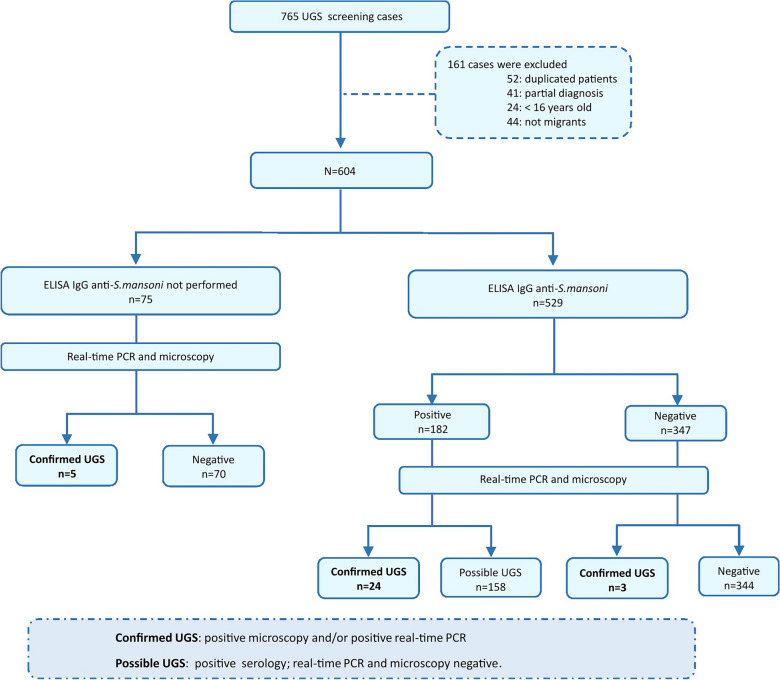

## Background

Schistosomiasis is a neglected tropical disease (NTD) endemic in more than 78 countries located in the tropical and subtropical regions of Asia, America and Africa. More than 700 million people live in at-risk areas, particularly affecting the poorest populations without access to appropriate sanitation systems and safe drinking water, and with limited access to health services [1]. The World Health Organisation (WHO) estimates that about 236 million people worldwide are currently infected mainly in sub-Saharan Africa, where it accounts for most of the annual incidence (> 90%) [2]. The number of people with chronic sequelae after infection is estimated at 440 million [3, 4].

The genus *Schistosoma* includes the species *Schistosoma mansoni, S. japonicum, S. intercalatum, S. guineensis* and *S. mekongi*, responsible for hepato-intestinal schistosomiasis, and *S. haematobium*, responsible for the urogenital schistosomiasis (UGS) form [4, 5]. UGS is mostly endemic in Africa and the Middle East [1] with clinical presentation varying between endemic area residents and international travellers [6]. It is usually asymptomatic, but the chronic form is associated with renal damage, fibrosis of the bladder and ureters, and an increased risk of developing squamous cell bladder carcinoma. In women, urogenital schistosomiasis can cause genital lesions and vaginal symptoms, while in men, it may affect the prostate and seminal vesicles. Long-term complications include infertility [1, 3, 7].

According to the WHO, the gold standard method for UGS diagnosis is the microscopic visualization of *S. haematobium* eggs in urine samples [1, 8]. This is a low-cost and specific technique; however, its sensitivity is generally low, especially for detecting a very low density of parasites, and it is affected by daily variations in egg excretion [9, 10].

Molecular techniques detecting *S. haematobium* DNA, such as the polymerase chain reaction (PCR) assay and especially real-time PCR, have shown higher diagnostic sensitivity, enabling early diagnosis and treatment monitoring [11]. However, their use is limited in resource-limited areas because of the high cost, specialized equipment and trained personnel requirements [3, 12]. Serological assays are sensitive techniques that have proven particularly useful among travellers or migrants in low-endemic and low-prevalence settings. However, they cannot discriminate active infection from past exposure as antibodies may persist over time [13–15].

Various alterations in blood and urine laboratory parameters can be observed from the interaction between the *Schistosoma* parasite and the host’s immune system, and infected organs and tissues. Eosinophilia, leukocyturia and elevated IgE levels are usually detected [16]. In cases of UGS, haematuria is also frequently observed. The severity of these biological alterations can vary depending on the disease stage and the individual’s response to infection [17, 18].

Recent increases in international travel and migration have led to more schistosomiasis diagnoses in non-endemic areas of Europe, but data on the prevalence of schistosomiasis among migrants from endemic regions living in Europe are limited [19–21]. Notably, two cases of autochthonous UGS transmission due to *S. haematobium* have been reported in Corsica (France) and Almeria (Spain) [22, 23].

This study addresses the need to assess and evaluate diagnostic methods for UGS in non-endemic settings, where accurate diagnosis remains challenging, particularly for asymptomatic migrants. Enhancing diagnostic accuracy is crucial for early detection and improved clinical management.

## Methods

### Study design and data collection

A retrospective cross-sectional study to assess the diagnostic performance of molecular and serological assays for imported UGS was conducted between November 2021 and December 2022. The study was coordinated by the Microbiology Department of Hospital Vall d’Hebron (HVH) and performed in the International Health Unit Vall d’Hebron-Drassanes (Barcelona). A total of 765 adult migrants from endemic regions were screened for UGS as part of a protocol designed for imported infectious diseases screening individuals from sub-Saharan Africa [24]. The following exclusion criteria were applied: (i) duplicate patient data, (ii) missing results for real-time PCR or microscopy, (iii) age < 16 years and (iv) travellers. Finally, 604 cases analysed by real-time PCR and microscopic examination were included in the study. Additionally, only 529/604 cases could be tested by an enzyme-linked immunosorbent assay (ELISA). The results of the complementary copro-parasitological analysis were collected in certain cases when performed as part of general clinical parasitological screening at HVH.

Sociodemographic and laboratory data, such as leukocyturia, microhaematuria, eosinophil count and serum IgE levels, were collected when available. Leukocyturia was defined as > 30 cells/µl; microhaematuria was classified as mild (30–100 cells/µl), moderate (100–500 cells/µl) and severe (> 500 cells/µl); eosinophilia was defined as > 7% eosinophils. Total serum IgE levels were also recorded, with values > 500 IU/ml considered hyper-IgE.

**Fig. 1:**
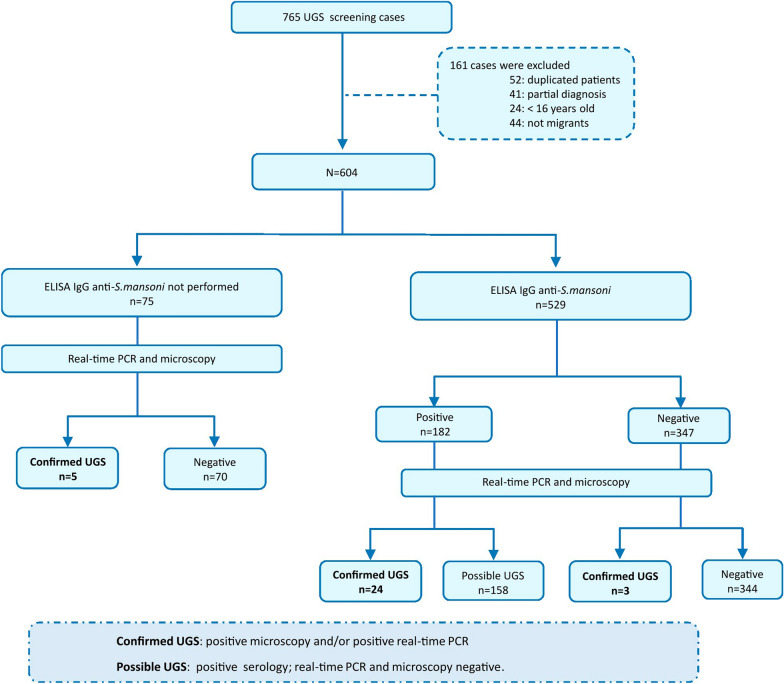
Flowchart of the study design detailing the methodological sequence of urine sample collection and analysis by conventional microscopy, real-time PCR and serology. UGS: urogenital schistosomiasis

A comprehensive flowchart of the study design, detailing the sequence of urine sample collection and analysis using conventional microscopy and real-time PCR, is included in Fig. 1.

### Case definition

Cases of UGS were defined as follows: (i) “positive confirmed case”: *S. haematobium* eggs in urine samples detected by microscopy visualisation and/or *S. haematobium* DNA detection by real-time PCR with no previous registered treatment; (ii) “possible case”: only positive serology with no detection of *S. haematobium* eggs in urine by microscopy or DNA by real-time PCR.

### Diagnostic methods

#### Conventional microscopy examination

Microscopic examination of urine samples was performed following WHO sample observation statements for *S. haematobium* diagnosis through concentration techniques [1]. A minimum of 10 ml urine, after sedimentation, was collected and concentrated at 423G for 5 min for microscopy examination. Stool examination is also included in the screening of asymptomatic migrants. It was carried out using Ritchie’s formalin-ether technique, and species identification was based on egg morphology [24].

#### Nucleic acid extraction and real-time PCR

DNA was extracted using the QIAsymphony SP (Qiagen GmbH, Hilden, Germany). DNA was extracted from 1 ml of centrifuged urine samples and eluted in 110 µl of the elution buffer, according to the manufacturer’s instructions.

A duplex real-time PCR targeted to the 121-bp tandem repeat Dra1 sequence of *S. haematobium* and the human RNase P gene (Taq Man Human RNase P detection reagent; Applied Biosystems, Foster City, CA) was performed according to Keller et al. [25], with some modifications. The primer sequences used were Sh_F 5′-GATCTCACCTATCAGACGAAAC-3′ and Sh_R 5′-TCACAACGATACGACCAAC-3′, and the probe sequence was Sh_P 5′-(FAM)-TGTTGGTGGAAGTGCCTGTTTCGCAA-BHQ1–3′. The final conditions in the PCR mixture were 1x QuantiTec Multiplex PCR kit (Qiagen), 160 nM of both primers for *S. haematobium* target, 80 nM of the TaqMan *S. haematobium* probe and 0.8x RNase P detection reagent. Reactions were performed using 5 µl of eluted DNA in a final volume of 25 µl. Amplifications were carried out in a CFX96 Touch Real-Time PCR Detection System (Bio-Rad, Hercules, CA).

In all cases, a sample was considered positive for *S. haematobium* DNA when the threshold cycle (Ct) for the *S. haematobium* target was < 40. A sample was considered invalid when the internal control (human RNase P gene) was inefficiently amplified. A positive control (*S. haematobium* DNA extracted from the urine of a patient previously diagnosed with UGS outside the study) and a negative control (nuclease-free water) were included in each real-time PCR run.

#### Serological assay

An enzyme-linked immunosorbent assay (ELISA) detecting IgG against *S. mansoni* (Novagnost *S. mansoni* IgG, Siemens Diagnostics, Marburg, Germany) was performed in serum samples following the manufacturer's instructions. Results are expressed as an index and considered positive when > 1.1.

### Statistical analysis

All data were analysed using RStudio (R version 4.1.1.) and SPSS Statistics 25.0. Qualitative variables were expressed as absolute frequencies and percentages. For proportion comparisons, bivariate analysis was carried out using the χ2 or Fisher’s exact test for frequencies < 5%, as appropriate. Differences were considered statistically significant if the *p*-value was < 0.05. Cohen’s kappa coefficient was used to measure the agreement between each test.

## Results

### Sociodemographic characteristics of study participants

A total of 604 cases tested for *S. haematobium* by both real-time PCR and microscopy in urine samples were included in the study. Most patients were men (87.3%), and the median age was 19 (range, 16 to 77) years: 327 of 604 (54.1%) patients were < 20 years old, 143 (23.7%) were between 21 and 29 years, 96 (15.9%) were between 30 and 50 years, and 38 (6.3%) were > 50 years.

### Urogenital schistosomiasis prevalence

UGS was confirmed in 32 out of 604 (5.3%) cases using both real-time PCR analysis and microscopy in urine samples. All *S. haematobium*-positive patients were male, with a higher proportion of positive patients (11/143; 7.7%) found in subjects aged 21–29 years. Almost all UGS-positive cases were observed in patients from West Africa: 11/32 (34.4%) from Mali, 9/32 (28.1%) from Gambia, 6/32 (18.8%) from Senegal, 4/32 (12.5%) from Guinea Bissau, 1/32 (3.1%) from Ghana, and 1/32 (3.1%) from Sudan.

### Diagnostic sensitivity of real-time PCR and microscopy assays

To evaluate the diagnostic accuracy of real-time PCR and microscopy, a reference standard test was established, which included UGS-positive results confirmed by real-time PCR and/or microscopy. In the entire study population, *S. haematobium* DNA was detected in 28/604 (4.6%) of cases, while *S. haematobium* eggs were observed in 24/604 (4%).

The diagnostic sensitivity values for each technique against the confirmed UGS diagnosis (32 positives out of 604) are shown in Table 1.

**Table 1:** Diagnostic sensitivity of real-time PCR and microscopy assays compared with the reference standard test (UGS-positive results confirmed by real-time PCR and/or microscopy)

	Positive result *n*/*n* total (%)	Sensitivity (95% CI)	Kappa coefficient (95% CI)
Real-time PCR	28/604 (4.6%)	87.5% (70.01–95.92)	0.93 (0.86–0.99)
Microscopy	24/604 (4%)	75% (56.25–87.87)	0.85 (0.75–0.95)

95% CI: 95% confidence interval; UGS: urogenital schistosomiasis

### Diagnostic accuracy of real-time PCR compared with microscopy

Taking microscopic visualisation as the reference method, the diagnostic performance of the real-time PCR assay is shown in Table 2. Discrepancies between the two tests were found in 12 of the 604 cases. Of these, eight were positive by real-time PCR and negative by microscopy, and in two of these eight samples, red blood cells (microhaematuria) were observed by microscopy. However, four samples were positive by microscopy and negative by real-time PCR; calcified eggs were observed in two of these four samples.

**Table 2:** Diagnostic performance of real-time PCR compared with microscopy

		Reference test: microscopy							
		+	−	Total	Sensitivity (95% CI)	Specificity (95% CI)	PPV (95% CI)	NPV (95% CI)	Kappa coefficient (95% CI)
Diagnostic testReal-time PCR	+	20	8	28	83.3 (61.81–94.52)	98.6 (97.19–99.36)	71.4 (51.13–86.05)	99.3 (98.11–99.78)	0.76 (0.62–0.89)
	−	4	572	576					
Total		24	580	604					

95% CI: 95% confidence interval; PPV: positive predictive value; NPV: negative predictive value; UGS: urogenital schistosomiasis

### Diagnostic accuracy of serology compared with real-time PCR and microscopy

The same reference standard test described previously was used to evaluate the performance of the serological assay. A total of 529 of the 604 cases included in the study underwent serological testing. The number of serology-positive cases obtained was 182/529 (34.4%), of which only 24/182 cases were confirmed by real-time PCR and/or microscopy examination. Comparing the serological technique with the reference standard, a low specificity and a low positive predictive value (PPV) were found. In addition, Cohen’s kappa coefficient indicated that the strength of agreement between techniques was slight (Table 3).

**Table 3:** Diagnostic performance values of serological testing compared with the reference standard test (UGS-positive results confirmed by real-time PCR and/or microscopy)

	Reference standard test UGS confirmed by real-time PCR and/or microscopy								
		+	−	Total	Sensitivity (95% CI)	Specificity (95% CI)	PPV (95% CI)	NPV (95% CI)	Cohen’s kappa coefficient (95% CI)
Diagnostic test serology	+	24	158	182	88.9 (69.70- 97.09)	68.5 (64.23- 72.53)	13.2 (8.80- 19.18)	99.1 (97.28- 99.78)	0.16 (0.09- 0.22)
	−	3	344	347					
	Total	27	502	529					

95% CI: 95% confidence interval; PPV: positive predictive value; NPV: negative predictive value; UGS: urogenital schistosomiasis

### Association between *S. haematobium* infection and leukocyturia, microhaematuria, eosinophilia and IgE levels

The correlation with certain clinical laboratory test results commonly used as indicators to estimate *S. haematobium* infection was assessed. In this study, a higher proportion of confirmed cases exhibited leukocyturia, microhaematuria, eosinophilia and elevated IgE levels in serum, showing statistical significance with the presence of UGS (*p* < 0.001) (Table 4).

**Table 4:** Association between laboratory test results and *Schistosoma haematobium* infection

		*n*	Confirmed UGS *n* (%)	*p*-value
Leukocyturia *n* = 532	Positive	19	5 (26.3)	< 0.001
	Negative	513	22 (4.3)	
Microhaematuria *n* = 532	High	1	0 (0)	< 0.001
	Moderate	5	4 (80)	
	Mild	4	2 (50)	
	Negative	522	21 (4)	
Eosinophilia *n* = 530	Positive	104	15 (14.4)	< 0.001
	Negative	426	12 (2.8)	
Elevated IgE levels *n* = 516	Positive	122	17 (13.9)	< 0.001
	Negative	394	9 (2.3)	

Leukocyturia was determined as > 30 cells/µl, microhaematuria was classified as mild (30–100 cells/µl), moderate (100–500 cells/µl) and severe (> 500 cells/µl); eosinophilia was defined as > 7% eosinophils, and elevated IgE levels were defined as total serum IgE levels > 500 IU/ml. UGS: urogenital schistosomiasis

A total of 89 out of 104 cases positive for eosinophilia were negative for UGS. Of these, 17 patients had helminth infections other than *S. haematobium* (Table 5). Notably, *S. mansoni* was the most common infection, reported in 8 out of 17 cases (47.1% of the total).

**Table 5:** Microscopic stool examination in eosinophilia-positive patients without confirmed UGS

		Eosinophilia-positive and UGS-negative *n* = 89, *n* (%)
Helminths n = 17	*Schistosoma mansoni*	8 (9)
	Coinfection *Schistosoma intercalatum/S. guineensis*-Trichuris trichura	1 (0.9)
	Hookworm (*Ancylostoma duodenale, Necator americanus*)	4 (3.6)
	*Ascaris lumbricoides*	3 (2.7)
	*Taenia* sp.	1 (0.9)
	Protozoa	12 (10.7)
	Negative	42 (37.4)
	No data	18 (16)

UGS: urogenital schistosomiasis

## Discussion

Imported schistosomiasis is increasing in non-endemic countries primarily because of rising global migration and increased travel to endemic regions in addition to the increased implementation of diagnostic techniques, mainly serological. However, there are limited data on its prevalence among migrants and travellers in Europe [19, 26, 27]. In our study, the prevalence of confirmed *S. haematobium* infection, using real-time PCR and/or microscopy, among migrants coming from schistosomiasis-endemic regions was 5.3%. The total prevalence including possible and confirmed UGS ranges from 7.5% to 43%, depending on the diagnostic method used [9, 28]. The highest prevalence rates are observed using serological tests [15, 29, 30]. The prevalence of imported UGS in low-endemic countries, based on urine samples examined by microscopy, is estimated at 5.7% among migrants from Africa and 6.8% from Sub-Saharan Africa [21]. Differences in prevalence may be attributed to migration patterns, demographic characteristics of the studied population or the diagnostic technique employed. Our study included asymptomatic adults > 16 years old, and urine samples were examined by microscopy visualisation and real-time PCR.

All confirmed cases of UGS were found in young males, predominantly from Mali (34.4%), followed by Gambia (28.1%) and Senegal (18.8%). This distribution aligns with other studies on imported infectious diseases [31–33]. For instance, previous research on imported schistosomiasis in Spain reported that most confirmed cases originated from Mali (50.3%), Senegal (22.7%) and Guinea-Bissau (7.4%) [34].

Microscopy, the reference method for diagnosing UGS, has been shown to have low sensitivity, particularly in cases of low-burden infections or in areas of low prevalence, such as non-endemic areas [32, 35, 36]. PCR assays are highly sensitive and specific in detecting DNA from a wide range of parasites, including *Schistosoma* spp. This makes them a valuable tool for detecting and identifying parasitic infections [37, 38]. Although there is a significant limitation, the actual infection status of individuals with negative real-time PCR and negative microscopy results remains unknown. The diagnostic sensitivity and PPV observed in this study for real-time PCR were lower than expected compared with other studies performed in endemic areas [39]. However, the diagnostic specificity obtained and NPV were higher than expected and concord with previous studies [25]. In the study by Keller et al. [25], real-time PCR outperformed egg detection by microscopy; however, the origin of the samples differed, and the expected density of parasites in endemic areas is higher than in non-endemic areas. The difference in specificity values could be justified by the discordant negative results they reported but could not explain.

Discrepancies between real-time PCR and microscopy, considered the reference test, were found in 12 out of 604 cases. In two samples negative by real-time PCR but positive by microscopy, microscopic examination revealed altered egg forms suggestive of calcified eggs, which may explain the failure to detect *S. haematobium* DNA. This finding could also be explained by the low amount of parasitic DNA present in the sample, particularly in patients in the chronic stage of the disease [40]. However, negative microscopy results were also reported in real-time PCR-positive samples. This may be attributed to the low sensitivity of the microscopy technique, affected by day-to-day variations in egg excretion, as well as to low infection intensities [10].

Patients arriving from schistosomiasis-endemic areas or suspected of infection are routinely screened using serological tests; however, the clinical interpretation of serological results remains challenging. These tests cannot differentiate between active, past or reinfection cases. Additionally, cross-reactivity with other helminth infections can lead to false-positive results. The presence of hybrids between human and animal schistosomes may also reduce detectable antibody responses. Furthermore, individuals with low antibody levels or delayed seroconversion may result in misdiagnosis [41]. A positive result is usually sufficient to prescribe treatment with praziquantel, a well-tolerated treatment that prevents serious long-term complications, especially when dealing with a potential loss of follow-up population, such as migrants [10, 42]. In this study, the serology used was an ELISA based on *S. mansoni* antigens; previous studies have shown that *S. mansoni* proteins used for serological testing have sufficient similarity to enable the detection of *S. haematobium* infections. However, the sensitivity of the test could be compromised [15, 27]. Compared with the reference standard, serology showed high sensitivity and a high negative predictive value; however, the specificity was notably low, resulting in a low PPV. These findings suggest that the ELISA technique is suitable for initial screening of a high-risk population in a non-endemic setting; therefore, it should be followed by a second-line diagnostic tool to confirm active infection [15, 43]. Given the large number of possible cases obtained, we can assume that there is overtreatment with praziquantel among the migrant population diagnosed with schistosomiasis by serological testing. Our results highlight the need for further in-depth studies to improve serological methods.

Leukocyturia, microhaematuria, eosinophilia, and elevated levels of IgE in serum are commonly used as indirect indicators for the estimation of *S. haematobium* infection [16, 44]. In this study, these biological markers were reported in many confirmed UGS cases, supporting their utility in suggesting schistosomiasis. The presence of leukocyturia was significantly associated with UGS, while microhaematuria remains the main sign of UGS, with a correlation of > 98% in endemic regions [45, 46]. Eosinophilia is a common finding in migrants from tropical areas and it may emerge in a wide range of disorders. Helminthiases are the most frequent cause of eosinophilia worldwide [47]. In the present work, UGS was found in 14.4% of eosinophilia-positive patients, and 19.1% were infected by other helminths, primarily *S. mansoni*. This proportion of eosinophilia associated with UGS is very similar to that found in similar studies from the same centre (12.5% relative eosinophilia in patients infected with *S. haematobium*). The same applies to elevated IgE levels, found in 13.2% of patients in previous studies [32]. Importantly, it should be noted that these clinical parameters are not specific to *S. haematobium* infection, and it may also be attributable to other infections or aetiological agents.

This study has some limitations due to its retrospective nature and reliance on previously recorded data, which resulted in some missing clinical and/or epidemiological data from some patients. Overall, this study represents a significant advance towards understanding the prevalence of imported UGS in migrant populations from endemic countries in non-endemic settings. The findings highlight the need for standardised diagnostic approaches to facilitate the management of this imported disease and support global efforts to control and eliminate this NTD.

## Conclusions

The results underscore the need for a reliable, standardised algorithm for UGS screening and diagnosis in non-endemic regions. Real-time PCR showed superior sensitivity over conventional microscopy for UGS diagnosis in a non-endemic setting. Nonetheless, microscopy remains widely used, especially in resource-limited settings. The agreement between the two methods is good, underscoring their utility in different contexts, considering factors such as microscopist experience, real-time PCR feasibility and the influx of migrant populations from endemic areas.

Additionally, serology is limited in confirming the diagnosis of UGS because of its low specificity. This study also found that leukocyturia, microhaematuria, eosinophilia and elevated serum IgE levels were recorded in a significant proportion of confirmed UGS cases, suggesting their potential as additional markers for clinical assessment.

## Data Availability

All datagenerated or analysed during this study are within the manuscript.
